# A Rare Cause of Portal Hypertension: Behcet’s Disease and Nodular Regenerative Hyperplasia of the Liver

**DOI:** 10.7759/cureus.1125

**Published:** 2017-03-29

**Authors:** Cyriac Philips, Rajaguru Paramaguru, Lijesh Kumar, Padmanabha Shenoy, Philip Augustine

**Affiliations:** 1 Hepatology and Liver Transplant Medicine, PVS Memorial Hospital; 2 Pathology, PVS Memorial Hospital; 3 Interventional Radiology, PVS Memorial Hospital; 4 Rheumatology, Care Clinics, Sree Sudheendra Medical Mission; 5 Gastroenterology, PVS Institute of Digestive Diseases, PVS Memorial Hospital

**Keywords:** portal hypertension, nrh, behcet’s disease, portosystemic shunt, erythema nodosum, fibrosis, cirrhosis, pathergy

## Abstract

Nodular regenerative hyperplasia (NRH) is a rare liver condition in which widespread benign transformation of the hepatic parenchyma into small regenerative nodules occur, leading to development of non-cirrhotic portal hypertension. Conditions associated with NRH include rheumatologic, hematological, autoimmune, infectious, neoplastic, or drug-related etiology. Accurate diagnosis is made on liver biopsy, showing diffuse micronodular transformation in the absence of fibrosis. Here, we present the second case in world literature of a middle-aged man presenting with recent onset sleep reversal and memory loss with darkening of skin for four years associated with systemic manifestations, in whom porto-systemic shunt syndrome due to NRH secondary to Behcet’s disease was eventually diagnosed.

## Introduction

Nodular regenerative hyperplasia (NRH) is an uncommon cause of non-cirrhotic intrahepatic portal hypertension. Micro vasculopathy and intrahepatic hypercoagulable state have been implicated in progressive hepatic architectural changes leading to NRH. Nodular regeneration of the liver was first defined by Steiner in 1959 characterized by small regenerative nodules distributed evenly throughout the liver in the absence of fibrosis leading to sinusoidal portal hypertension (PHT) [1-2]. Behçet’s disease (BD) is a rare systemic vasculitis disorder of unknown etiology in which recurrent/relapsing attacks of oro-genital aphthae and ocular manifestation in the form of uveitis occur with varied systemic involvement. The relapses are unpredictable and can include severe manifestations affecting the cardiovascular, central nervous, and gastrointestinal systems. The disease typically appears between the third and the fourth decades of life, and treatment options including systemic anti-inflammatory and/or immune modulating agents are mostly aimed at relief of symptoms and for control of disease progression. The systemic vasculitis of BD affecting the hepatobiliary system very rarely leads to NRH and portal hypertension and has been reported only once before [3-4]. We describe a middle-aged man who was referred for management of cirrhosis and its complications, in whom non-cirrhotic PHT secondary to NRH associated with BD was diagnosed. This case emphasizes the importance of active investigational search for treatable causes of PHT and of non-cirrhotic origin that can help in better prognostication of affected patients.

## Case presentation

A 40-year-old man, who was a known case of systemic hypertension for 13 years, was referred to our liver clinic for evaluation of progressive darkening of skin over the face for four years and altered sleep pattern for six months associated with recent onset intermittent memory loss. He did not smoke or abuse alcohol and had no family history of liver disease. Six years ago, he started noticing recurrent painful oro-mucosal ulcerations that lasted for few weeks and were self-limiting. These were sometimes associated with painful ulcers over the glans penis that were self-limiting too.

Two years ago, he developed recurrent crops of reddish nodular lesions over both legs that healed with hyperpigmentation leading to extensive darkening of the skin and scarring over the anterior aspect of the lower limbs. There were no symptoms associated with ocular, central or peripheral nervous and gastrointestinal systems. Since the last four years, the patient had been noticing darkening of the skin over the face and his wife started noticing that the patient was being forgetful. His sleep pattern was affected with difficulty in falling asleep and recurrent nocturnal awakenings. For these symptoms, he visited a physician who ran some tests and performed an ultrasound imaging of the abdomen, diagnosing him to have chronic liver disease and grade one hepatic encephalopathy. He was then referred to a specialist who diagnosed him with non-alcoholic fatty liver disease related cirrhosis with hepatic encephalopathy; upper gastrointestinal endoscopy done at the time did not reveal varices. In view of raised arterial ammonia (168 μmol/l, normal <47 μmol/l), he was prescribed ammonia lowering therapy. The patient’s sleep patterns improved substantially, but the oro-mucosal ulcers recurred with new onset leg edema after which he was referred to our center.

**Figure 1:**
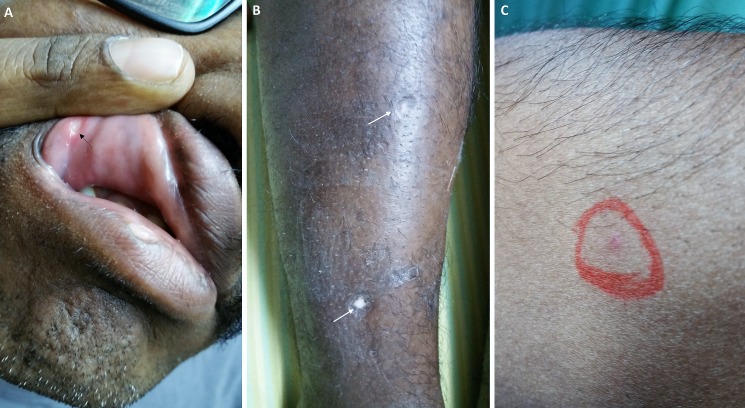
Clinical examination findings Clinical examination showing active oro-mucosal aphthous ulcers (A); hyperpigmentation and scarring over both legs with areas of pitting and tender nodularity (B); a pathergy test was positive at 36 hours (C).

On evaluation at our center, the patient was alert to person and place, but not to time. Pallor was present, without icterus, lymphadenopathy or clubbing. Facial skin hyperpigmentation was evident with active aphthous ulcers in the oral cavity (Figure 1A). Hyperpigmentation and scarring over both legs with areas of pitting and tender nodularity were seen (Figure 1B). An abdominal examination revealed a firm palpable liver 3 cm below the right costal margin without splenomegaly or ascites. Complete hemogram was suggestive of anemia (10.2 g/L, normal 13 to 15 g/L), thrombocytopenia (90 x 10^9^/L, normal 150 to 400 x10^9^/L) and high erythrocyte sedimentation rate (90 mm in the first hour); a liver function test revealed hypoalbuminemia and albumin:globulin ratio reversal; electrolytes, prothrombin time, activated partial prothrombin time and renal function tests were normal. In view of recurrent oro-mucosal and genital ulcerations associated with suspicious erythema nodosum or cutaneous manifestation of polyarteritis nodosa, blood for antinuclear antibody, anti-smooth muscle antibody, anti-liver kidney microsomal antibodies, rheumatoid factor, anti-cardiolipin, lupus anticoagulant tests, antibodies to hepatitis C, hepatitis B and HIV was sent; all non-contributory. However, the pathergy test was positive at 36 hours (Figure 1C).

**Figure 2:**
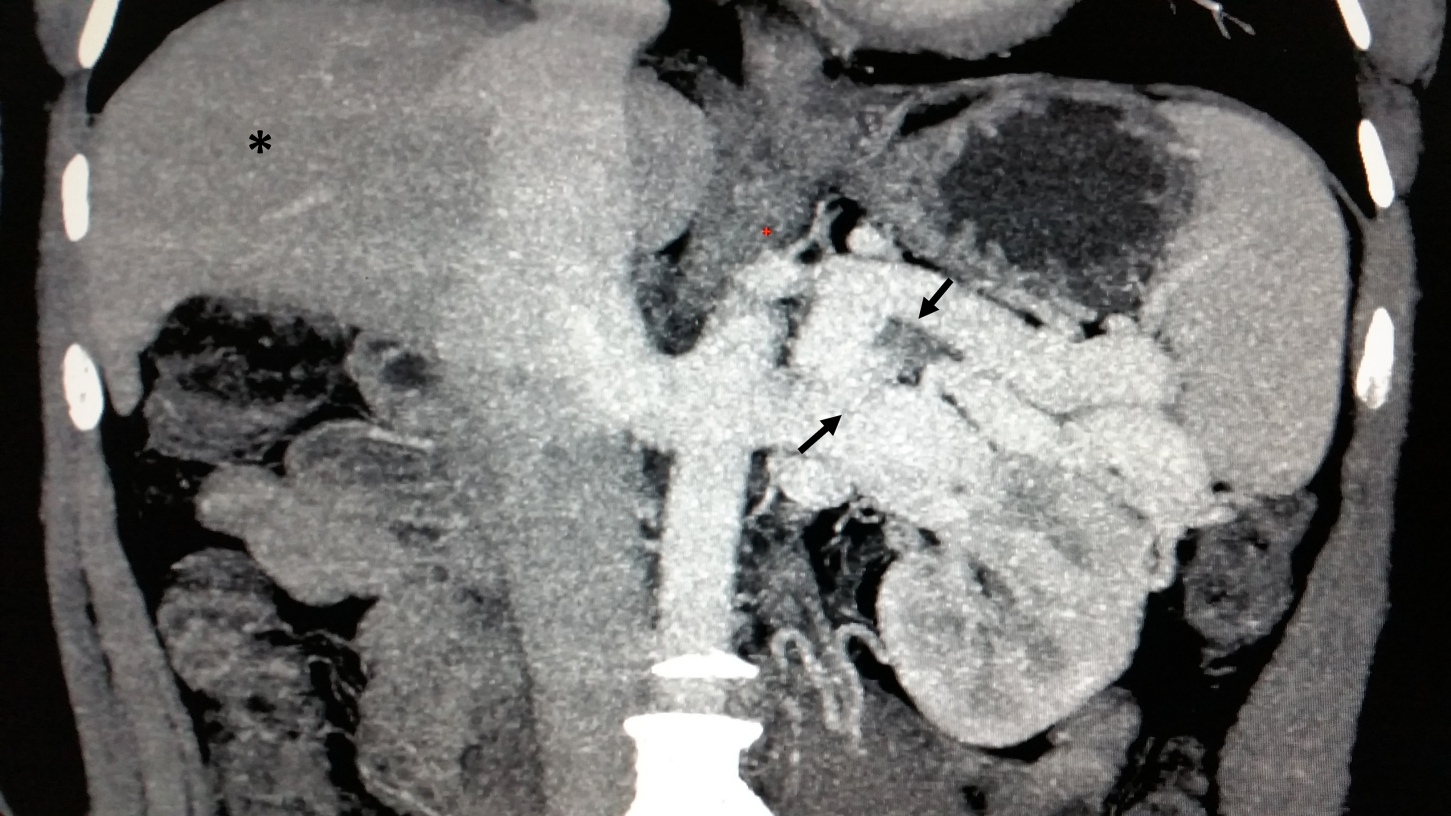
Computed tomographic venogram of the abdomen A computed tomographic venogram of the abdomen showing cirrhotic architecture of the liver (asterisk) and associated large tortuous lieno-renal-shunt (arrows).

A computed tomography (CT) of the abdomen revealed cirrhotic architecture of the liver (Figure 2, asterisk) and associated large tortuous lieno-renal-shunt (Figure 2, arrows) without associated portal vein thrombosis or splenomegaly.

**Figure 3:**
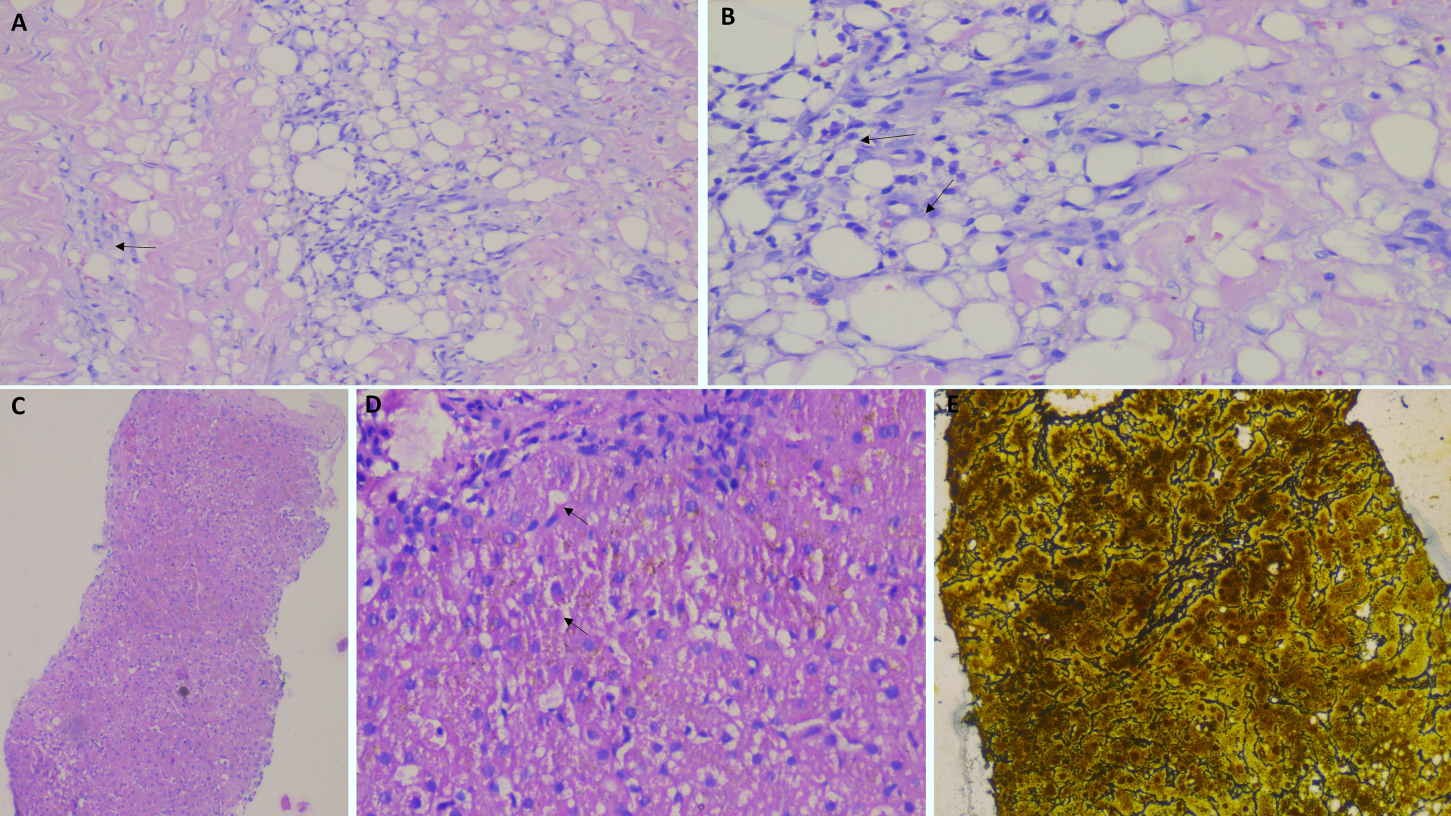
Histopathological findings Biopsy from the skin lesions over the leg showing sub-epithelial perivascular lymphoplasmacytic cell infiltrate with scattered macropahges (A, arrow; 20X, Hematoxylin and Eosin stain); associated foci of panniculitis (B, arrows; 40X, Hematoxylin and Eosin stain) suggestive of chronic phase of erythema nodosum; liver biopsy showing distortion of lobular architecture with increased cord thickness of the hepatocytes, with nodule formation (C; 10X, Hematoxylin and Eosin stain); sinusoidal dilatation and congestion (D, arrows; 40X, Hematoxylin and Eosin stain) in the absence of fibrosis; reticulin staining showed increased hepatocyte cord thickness (E, 40X)

A biopsy from the skin lesions over the leg showed sub-epithelial perivascular lymphoplasmacytic cell infiltrate with scattered macropahges (Figure 3A, arrow), occasional eosinophils and neutrophils with endothelial swelling associated with foci of panniculitis (Figure 3B, arrows) at lower part of dermis, suggestive of chronic phase of erythema nodosum. Portal hypertension was confirmed. However, a transient elastography of the liver showed normal stiffness (4.8 kilo Pascals). Non-cirrhotic portal hypertension was considered and a transjugular liver biopsy was performed with hepatic venous pressure gradient measurement (HVPG). The HVPG was 8 mm Hg (normal < 6 mm Hg), and the liver biopsy showed distorted lobular architecture with increased cord thickness of the hepatocytes with nodule formation (Figure 3C); sinusoidal dilatation and congestion (Figure 3D, arrows) in the absence of fibrosis. Reticulin staining showed increased hepatocyte cord thickness (Figure 3E) and trichrome stains showed absence of fibrosis, features that were suggestive of NRH.

The patient fulfilled Behcet's syndrome International Study Group (ISG) criteria and the recently proposed International Criteria for Behçet's Disease (ICBD) for diagnosis of BD; PHT was diagnosed secondary to NRH with hepatic encephalopathy due to portosystemic shunt syndrome. Ammonia lowering therapies were optimized, and low dose corticosteroids were initiated along with tacrolimus. The patient has been clinically well four weeks after therapy initiation, and portosystemic shunt closure is planned in the event of medically refractory or recurrent hepatic encephalopathy.

## Discussion

Behcet’s disease is proposed to originate from an autoimmune response that may be triggered by infectious or other environmental agents in genetically susceptible individuals with progressive vasculitic and proinflammatory affliction of affected organ systems. Behcet’s disease is diagnosed on clinical grounds as per ISG or ICBD criteria. In the former, oral aphthosis is mandatory and two other clinical features from the four remaining (genital aphthosis, skin manifestations such as erythema nodosum, eye involvement in the form of uveitis and positive pathergy test) are necessary to diagnose BD. In ICBD, ocular lesions, oral aphthosis and genital aphthosis are each assigned two points, with cutaneous, central nervous system, vascular manifestations and positive pathergy test one point each. A patient scoring ≥ four points is classified as having BD. HLA-B51 and HLA-B5701 are strongly associated with disease pathogenesis, most notably in populations along the Old Silk Route, but HLA typing is not necessary for diagnosis [5-6]. NRH is thought to result from an intra-hepatic vasculopathy in association with systemic diseases that include vasculitis processes. This vasculopathy leads to microvascular perfusion resulting in hepatocyte injury and hepatocyte regeneration. The latter is associated with nodule formation that compresses surrounding hepatic parenchyma as well as the portal and central veins leading to portal hypertension and its complications. NRH mimics cirrhosis; both exhibit macroscopic multi-nodularity. However, the former lacks fibrosis and intrahepatic vascular shunts seen in the latter [7-8]. In BD, the most commonly described cause of PHT is Budd-Chiari syndrome secondary to thrombophlebitis of the hepatic veins [9]. Bloxham and colleagues were the first to report an association between BD and NRH in 1992. In their report, a 67-year-old male with clinical features of BD and dying of myocardial infarction and small bowel gangrene was diagnosed to have NRH on postmortem biopsy [10].

Our case is unique in many aspects. This is the second report in the world associating BD and NRH, after the initial report in the year 1992. Our patient was diagnosed while alive contrary to the first case where diagnosis was postmortem. Our patient did not present with classical features of PHT such as ascites or variceal bleeding. We attribute this to the large portosystemic shunt that helped decompress the portal system. However, the patient did have clinical features to suggest portosystemic shunting (Type B hepatic encephalopathy). Behcet’s disease is classically diagnosed clinically, as was seen in our case who also mounted a positive pathergy response (insertion of a small sterile needle intradermally leading to occurrence of a small red papule or pustule at the site within forty eight hours constitutes a positive result, indicating that the immune system is overreacting to a minor injury). Nonalcoholic fatty liver disease is one of the most important etiologies of chronic liver disease globally and quite frequently diagnosed in the absence of other known etiologies. However, one must not be in haste to diagnose a patient with chronic liver disease. In our patient, the absence of common complications of cirrhosis such as ascites, jaundice and/or variceal bleeding was evident from history. That, coupled with other systemic manifestations not explained by liver disease alone prompted us to refine our diagnostic needs. A cirrhotic-looking liver is not always cirrhotic. Transient elastrography is a well validated tool for diagnosis of advanced fibrosis and cirrhosis, which was very helpful in decision making regarding a liver biopsy that ultimately shed light on the cause of PHT. Patients who present with uncommon manifestations of PHT associated with systemic symptoms and signs unexplained by liver disease alone, need to be astutely evaluated, guided by clinical decision making.

## Conclusions

We describe the second case of PHT secondary to NRH in a patient with Behcet’s disease in world literature. However, the first described case was diagnosed postmortem. We urge clinicians to look beyond the ordinary while diagnosing etiologies of liver disease in patients who present with atypical complications of cirrhosis and/or PHT and/or unexplainable associated systemic symptoms and signs.
